# Lamivudine Concentration in Hair and Prediction of Virologic Failure and Drug Resistance among HIV Patients Receiving Free ART in China

**DOI:** 10.1371/journal.pone.0154421

**Published:** 2016-04-27

**Authors:** Jing Yan, Jia Liu, Bin Su, Xiaohong Pan, Zhe Wang, Jianjun Wu, Jiafeng Zhang, Yuhua Ruan, Jenny Hsi, Lingjie Liao, Yiming Shao, Hui Xing

**Affiliations:** 1 State Key Laboratory of Infectious Disease Prevention and Control (SKLID), National Center for AIDS/STD Control (NCAIDS) and Prevention, Chinese Center for Disease Control and Prevention (China CDC), Collaborative Innovation Center for Diagnosis and Treatment of Infectious Diseases, Beijing, China; 2 Henan Center for Disease Control and Prevention, Zhenzhou, Henan Province, China; 3 Anhui Center for Disease Control and Prevention, Hefei, Anhui Province, China; 4 Zhejiang Center for Disease Control and Prevention, Hangzhou, Zhejiang Province, China; Shanghai Medical College, Fudan University, CHINA

## Abstract

**Background:**

The assessment of adherence to antiretroviral therapy (ART) is important in order to predict treatment outcomes. Lamivudine (3TC) is one of the most widely used NRTIs in China, but its concentrations in hair and association with virologic failure and drug resistance have not been studied.

**Methods:**

We conducted a cross-sectional survey to investigate 3TC concentrations in hair as a predictor of virologic failure and drug resistance among HIV patients receiving free ART. We also compared the capacity of hair 3TC concentrations with self-reported adherence in predicting virologic responses. Hair 3TC concentrations were detected through the LC-MS/MS system.

**Results:**

In patients without HIV drug resistance (HIVDR), with a threshold hair 3TC concentration of 260 ng/g, the sensitivity and specificity in predicting virologic suppression were 76.9% and 89.9%, respectively. Some factors, including CD4^+^ cell counts, initial treatment regimens with 3TC, and current regimens with second-line drugs, influenced the association between hair 3TC concentrations and virologic suppression. In patients who experienced virologic failure with HIVDR, with a threshold of 180 ng/g, the sensitivity and specificity were 70.0% and 74.4%, respectively. Hair 3TC concentrations had higher sensitivity and specificity in predicting virologic failure and drug resistance than self-reported adherence.

**Conclusions:**

The hair 3TC concentration was a stronger indicator than self-reported adherence in predicting virologic failure and drug resistance in HIV patients receiving free ART.

## Introduction

Highly active antiretroviral therapy (HAART) effectively reduces HIV patients’ morbidity and effectively prolongs their disease-free lives. According to a previous study, antiretroviral treatment outcomes are relevant to immunologic, pharmacokinetic factors, and adherence to regimens [1]. Strict adherence to regimens ensures better treatment outcomes, but suboptimal drug levels may lead to the emergence of drug-resistant HIV variants [2, 3]. Therefore, the assessment of adherence to ART is important to predict virologic outcomes, disease progression, and death.

Many methods are used to evaluate the adherence to antiretroviral therapies, such as self-reported questionnaires, pill counting, the Medication Event Monitoring System (MEMS), the visual analogue scale, and drug concentrations in plasma or urine [3, 4]. The advantages and disadvantages of these methods are obvious. Self-reported questionnaires and the visual analogue scale are often used in resource-limited countries because of their low cost and simplicity. However, both of these methods tend to overestimate patients’ adherence due to recall bias and social desirability [5–7]. Pill counts are not suggested to solely assess patients’ adherence [7]. In previous studies, though MEMS has shown good correlation with the HIV load, the estimation of adherence may be biased when assessed through MEMS [7, 8]. Measurement of drug concentrations in plasma and urine may be more sensitive indicators, however, both only provide the adherence within 1 to 2 days of sampling [9–11]. Alternatively, measuring drug concentrations in hair has the potential to provide an objective estimate of a long-term exposure, which will be more meaningful in predicting treatment outcomes and efficacy of ART [12]. In individual patients, hair growth varies from 0.5 to 1.5cm each month and drug concentrations in the hair root (hair 0.5–1.0 cm from the scalp) record the last month’s history of drug use. Thus a monthly history of drugs is recorded in the hair root[13–15]. Many studies showed that hair drug concentrations can strongly predict virologic outcomes [16–18].

The current recommended regimens in China include two nucleotide or nucleoside reverse transcriptase inhibitors (NRTIs) and one non-nucleoside reverse transcriptase inhibitor (NNRTI) or protease inhibitor (PI). Since 2008, China’s National Free Antiretroviral Treatment Program (NFATP) had changed its first-line ART regimen by replacing didanosine (DDI) with lamivudine (3TC) [19, 20]. So far, 3TC has been the most widely used NRTI in China. In another research conducted in China, researchers concluded that lamivudine (3TC) produced better treatment outcomes with a higher virologic suppression rate and lower drug resistant rate [21]. At the same time, researchers admitted that adherence to treatment regimens should be given more attention in the future. According to the pharmacokinetic study of NRTI drugs such as 3TC, d4T, and AZT, they need be biologically activated through intracellular conversion to phosphorylated metabolites [22]. Thus, if the NRTI plasma concentration has any association with the intracellular 3TC concentration or can predict treatment outcomes still remains uncertain [23]. What is more, another study showed that 3TC plasma concentrations among patients may be influenced by many factors, such as different durations between the sampling time and the last dose, the variability of the body mass index, and renal conditions [24]. The elimination half-life of 3TC is as short as 3–4 hours and this may contribute to a large variation in 3TC plasma concentrations [25, 26]. Considering the long-term assessment of regimen adherence and the complex testing of intracellular 3TC active metabolite concentrations, we conducted this study to find if hair 3TC concentrations can predict virologic failure and drug resistance, and also compare the capacity of hair 3TC concentrations with self-reported adherence in predicting virologic responses.

## Methods

### Ethics Statement

The IRB of Chinese Center for Disease Control and Prevention approved this study and the approval number was X120331207. We obtained written informed consents.

### Sample population and sample collection

We conducted a cross-sectional survey in HIV patients receiving the lamivudine regimen from 2013 to 2014. 287 patients were enrolled from Zhejiang, Anhui, and Henan Provinces. The inclusion criteria were: patients ≥ 18 years old, had received national free ART treatment for ≥12 months, current treatment regimen included lamivudine, and willingness and consent to participate. Patients who were treated with second-line regimens as the initial treatment were excluded. Basic information (age, sex, income per month, marital status, education, occupation, HIV transmission route, initial ART regiment, current ART regiment, duration of ART, missed dose in the past month, and ratio of on-time drug intake in the past month), blood specimens, and hair specimens for lamivudine were collected. Hair samples were collected from the occipital area as previously described because it has the lowest variability in hair growth rate [17, 27]. At least 30 strands of hair were cut as close to the scalp as possible and the distal portion labeled to denote directionality [28]. At least a 1 cm hair of sample was cut and at least 1cm of hair close to the scalp should be remained for testing. The hair specimens were placed in small plastic bags and were stored at room temperature in the dark to avoid excessive exposure to moisture. Blood samples were also collected for CD4^+^ cell count, HIV-1 RNA viral load testing, and genotypic drug resistance (HIVDR) testing as previously described [29, 30]. All the hair samples and blood samples were collected within the same hour. In accordance with the WHO survey guideline, successful viral suppression was defined as HIV RNA level <1,000 copies/mL using a quality-assured viral load assay. In samples with a viral load (VL) ≥1000 copies/mL, HIVDR genotyping was performed by in-house polymerase chain reaction (PCR). The study was approved by the institutional review board (IRB) at the National Center for AIDS/STD Control and Prevention of the China Center for Disease Control and Prevention (NCAIDS, China CDC).

### Hair ART quantification

The hair 3TC concentration was determined using liquid chromatography and tandem mass spectrometry (LC-MS/MS) at iPhase Pharma Services in Beijing, China. Briefly, 3TC concentrations in 1–1.5 cm hair samples near the scalp approximately represented the drug accumulation in the past month. Hair samples were cut into small segments, yielding approximately 2 mg of hair [31], and 50 μL of 50% methanol was added, and samples were whirled to blend well. Then 50 μL IS (0.02 μg/ml lamivudine-^15^N_2_,^13^C as an internal standard) and 0.9 ml methanol were added to each sample. Samples were shaken in a 37°C water bath overnight, followed by centrifugation at 13,000 rpm for 5 minutes. 500 μL supernatant was extracted and mixed with 500 μL deionized water, and then the mixture was whirled. 10 μL of the mixture was used for LC-MS/MS analysis.

The LC-MS/MS system consists of an Agilent 1200 Series pump (G1312A) and autosampler (G1367B), and an AB Sciex LC triple quadrupole tandem mass spectrometer. The mass spectrometer was set to electrospray ionization in a positive multiple reaction monitoring mode. The precursor/product transitions (m/z) were at 230.3 > 112.2 m/z for lamivudine and 233.2 > 115.3 m/z for the internal standard (IS) lamivudine-^15^N^2^, ^13^C. The declustering potential and collision energy were 30 V and 18 V, respectively, for lamivudine and IS. The source temperature was 550°C. The HPLC conditions were as follows: the column was an Agela venusil ASB C18, (50 × 4.6mm), 5 μm, 150Å, and the mobile phase was composed of methanol and HPLC-grade water. Elution was achieved with an isocratic flow of water:methanol (55:45, v:v) at a flow rate of 0.8 mL/min. Data processing was performed using Analyst 1.5 software.

The calibrated hair samples were prepared as follows: 2 mg of blank cut hair samples were placed into different test tubes, and 50 μL 0.4, 0.8, 2, 5, 20, 40, 64, or 80 ng/ml standard solutions was added. Then, these samples were treated in the same way as above. We controlled the relative standard deviation (RSD) of the standard curve at less than 15%. The linear range of the standard curve was 10–2000 ng/g.

### Statistical analysis

Questionnaires were double-entered and validated with EpiData 3.1 (The EpiData Association, Denmark) and then were analyzed by the Statistical Analysis System (SAS 9.2, SAS Institute Inc., Cary, NC, USA) and SPSS Statistics 19 (SPSS Inc., USA). Demographic variables were described with descriptive statistics and the Wilcoxon signed rank test was used to compare hair 3TC concentrations among the three groups for virologic suppression, virologic failure without HIVDR, and virologic failure with HIVDR. In order to explore the association between the 3TC concentration in hair and virologic suppression/drug resistance, we used multivariate logistic regression to calculate the odds ratios and 95% confidence intervals. At the same time, we selected cut-off concentrations with receiver operating characteristics. In addition to investigating factors which have an influence on the relationship between hair 3TC concentrations and viral load, we used stratified analysis through the Chi-square test or Fisher’s exact test to calculate the P value among different stratifications. Hypothesis testing was two-sided with α = 0.05.

## Results

A total of 287 patients from Zhejiang, Anhui, and Henan Provinces of China were enrolled in the survey. The characteristics of the subjects are shown in Table 1. The average age was 44.9±10.2 years, 52.6% were male, the proportion with an income per month of less than 1,000 Yuan was 74.6%, 76.0% were married, 19.5% had a high school or higher education level, and farmers accounted for 68.3%. The two major reported transmission routes were blood donations from former commercial plasma donations (former plasma donors were prople who acquired HIV infection through illegal hemapheresis in the mid-1990s in China.) and sexual intercourse, which accounted for 70.7% and 25.8%, respectively. The median duration of ART treatment was 87.4±39.2 months. The proportion of DDI-based initial ART regimens was 45.6%. 47.4% of current ART regimens were the second line of 3TC plus (AZT or TDF or d4T) plus LPV/r. Among these 287 patients, 79 (27.5%) had experienced virologic failure and 40 (13.9%) had drug resistant mutations.

**Table 1:** Characteristics of HIV patients receiving free ART in three provinces of China.

	Number	%
Total	287	100
Site		
Zhejiang	78	27.2
Anhui	56	19.5
Henan	153	53.3
Age (mean ± SD, year)	44.9±10.2	
Sex		
Male	151	52.6
Female	136	47.4
Income per month (RMB, yuan)		
0–1000	214	74.6
1000–2000	12	4.2
2000 or above	61	21.2
Marital status		
Married	218	76.0
Other	69	24.0
Education		
Illiterate	59	20.6
Primary school	90	31.3
Junior high school	82	28.6
High school or more	56	19.5
Occupation		
Farmer	196	68.3
Others	91	31.7
HIV transmission route		
Blood Donation	203	70.7
Sexual intercourse	74	25.8
Drug injection	3	1.1
Other	7	2.4
Initial ART regimen		
3TC+AZT+NVP/EFV	73	25.5
3TC+D4T+NVP/EFV	77	26.8
DDI +AZT+NVP/EFV	131	45.6
3TC+TDF+NVP/EFV	4	1.4
Others	2	0.7
Current ART regimen		
3TC+AZT+NVP/EFV	106	36.9
3TC+ D4T +NVP/EFV	31	10.8
3TC+ TDF+NVP/EFV	14	4.9
3TC+AZT/TDF/D4T+LPV/r	136	47.4
Duration of ART (mean±SD, month)	87.4±39.2	
Missed doses in the past month		
Yes	59	20.6
No	228	79.4
Ratio of on-time drug intake in the past month		
90% or more	244	85.0
75–90%	24	8.4
50–75%	7	2.4
<50%	12	4.2
HIV viral load (copies/ml) at the survey		
< 150 (detection limit)	158	55.1
150–1000	50	17.4
1000–10,000	30	10.4
≥ 10,000	49	17.1
Drug resistance at the survey*		
No	247	86.1
Yes	40	13.9
CD4 cell count (cells/mm^3^) at the survey		
<200	44	15.3
200–350	78	27.2
≥350	165	57.5

*Drug resistance was defined as viral load≥1000 copies/ml with drug resistant mutations.

**Fig 1:**
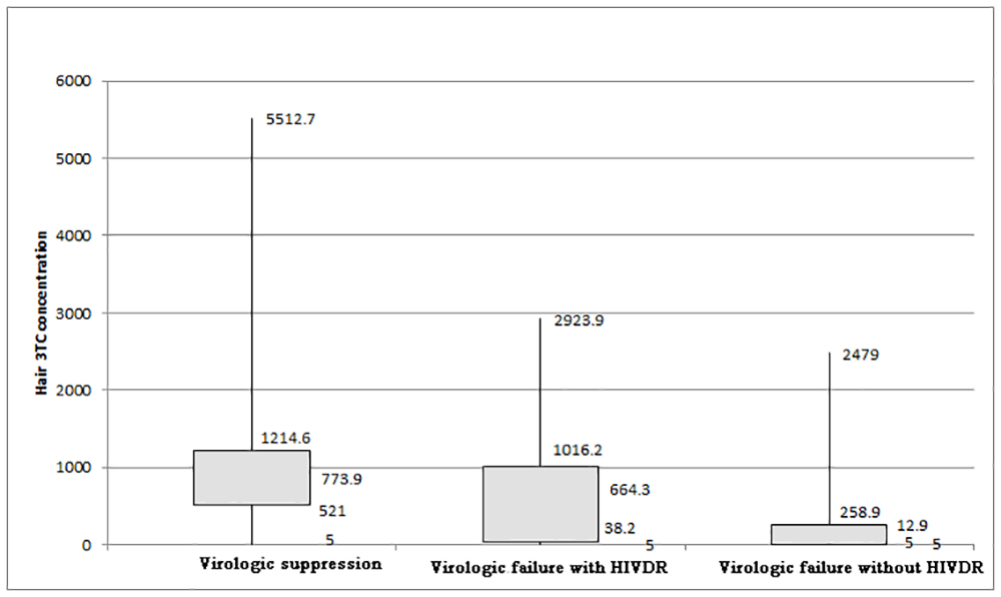
The box plot of hair 3TC concentration.

Hair 3TC concentrations among people are shown in Fig 1. We divided the 287 patients into three groups according to viral load and drug resistant mutations. There were 208, 39, and 40 patients in the groups of virologic suppression, virologic failure without HIVDR, and virologic failure with HIVDR, respectively. The mean hair 3TC concentrations in the groups of virologic suppression, virologic failure without HIVDR, and virologic failure with HIVDR were 915.0±670.5 ng/g, 284.1±538.9 ng/g, and 648.4±616.9 ng/g, respectively. The Wilcoxon signed rank test was used to compare the medians of hair 3TC concentrations among these three groups, and the difference among these groups were statistically significant (virologic failure with HIVDR vs. virologic suppression, p = 0.0125; virologic suppression vs. virologic failure without HIVDR, p<0.0001).

We then conducted a multivariate logistic regression model to examine the association of different indictors with the dichotomous outcome of virologic suppression after controlling for several variables, such as age, sex, income per month, marital status, education, occupation, HIV transmission route, initial ART regiment, current ART regiment, duration of ART, and CD4 count at the survey (S1 Table). The adjusted odds ratio of the 3TC concentration in hair was 11.5 (compared to 3TC concentration < 260ng/g, 95% CI 5.7, 23.2, p<0.001). Missing doses in the past month and the ratio of on-time drug intake in the past month were not included in the final model. What is more, after adjusting for demographic factors, only those with a higher 3TC contents in hair were observed to be at higher risk for emerging drug resistance than those with a lower 3TC contents (<180 ng/g)(AOR = 6.8, 95% CI 2.5,18.2, p<0.001).

Through the receiver operating characteristic curve (ROC), the optimal cut-off (that maximized the Youden Index) was 260ng/g. The sensitivity, specificity, positive predictive value, and negative predictive values were 54.4%, 89.9%, 67.2%, and 83.9%, respectively. The large amount of false-negatives (patients with HIVDR always tend to have higher 3TC concentrations while experiencing virologic failure) resulted in a lower sensitivity. Thus, we constructed another receiver operating characteristic curve (ROC) among patients without HIVDR, and the optimal cut-off was also approached at 260ng/g, with 76.9% sensitivity, 89.9% specificity, 58.8% positive predictive value, and 95.4% negative predictive value. The sensitivities and specificities at different hair 3TC concentrations are listed in Table 2. We also used interviewer-assisted questionnaires to assess the adherence in the past month, including missed doses in the past month and the ratio of on-time drug intake in the past month. When we used the threshold of hair 3TC concentration at 260ng/g, the hair 3TC concentration was a much stronger indicator than variables of self-reported adherence in predicting virologic failure. We examined whether demographic and treatment factors would influence the threshold of hair 3TC in predicting virologic failure. Stratified subgroups are listed in S2 Table. The Chi-square test or Fisher’s exact test was used to calculate the P value among different stratifications. Sensitivity, specificity, positive predictive value, and negative predictive values were not significantly affected by most stratified factors, except for CD4^+^ cell counts, initial ART treatment, and current ART treatment. Patients who had higher CD4^+^ cell counts (≥350 cells/ml) had lower positive predictive values (77.8%, 77.8%, and 37.5% for <200 cells/ml, 200–350 cells/ml, and ≥350 cells/ml, respectively, p = 0.01) and higher negative predictive values (89.5%, 89.6%, and 98.4% for <200 cells/ml, 200–350 cells/ml, and ≥350 cells/ml, respectively, p = 0.01). What is more, we found that patients who had received 3TC as the initial ART treatment regimen had higher sensitivity (91.7% vs. 53.3%, p = 0.02). Receiving second-line ART treatment may lower the negative predictive values (91.9% vs. 98.2%, p = 0.04).

**Table 2:** Optimizing the threshold of hair 3TC concentration and comparing it with self-reported adherence among HIV patients without HIVDR (N = 247).

	VL≥1000	VL<1000	Sensitivity	Specificity	PPV	NPV	Youden Index
Hair 3TC<100	25	14					
≥100	14	194	64.1%	93.3%	64.1%	93.3%	57.4%
Hair 3TC<200	29	20					
≥200	10	188	74.4%	90.4%	59.2%	94.9%	64.7%
Hair 3TC<260	30	21					
≥260	9	187	76.9%	89.9%	58.8%	95.4%	66.8%
Hair 3TC<300	30	25					
≥300	9	183	76.9%	88.0%	54.5%	95.3%	64.9%
Hair 3TC<400	30	40					
≥400	9	168	76.9%	80.8%	42.9%	94.9%	57.7%
Hair 3TC<500	30	51					
≥500	9	157	76.9%	75.5%	37.0%	94.6%	52.4%
Missed dose in past month							
Yes	15	33					
No	24	175	38.5%	84.1%	31.3%	87.9%	22.6%
Ratio (90%) of on-time drug intake in the past month							
Yes	13	24					
No	26	184	33.3%	88.5%	35.1%	87.6%	21.8%

The threshold of hair 3TC concentrations associated with virologic failure with HIVDR is presented in Table 3. According to the receiver operating characteristic curve (ROC) among 79 HIV patients with a viral load≥1000 copies/ml, the optimal operating point on the ROC curve was 180ng/g with the area under the ROC curve of 0.72. This threshold corresponds to a sensitivity of 70.0%, a specificity of 74.4%, a positive predictive value of 73.7%, and a negative predictive value of 70.7%. The threshold of 180ng/g had a more balanced sensitivity and specificity than 300ng/g with the same Youden Index. Although hair 3TC concentrations had a lower sensitivity than self-reported adherence in predicting HIVDR, its specificity was an obvious advantage and the Youden Index was also higher (Table 3). Through the stratified analysis to explore factors which may influence the association between hair 3TC concentrations and the emergence of drug resistance (S3 Table), we found that 3TC in the initial treatment regimens only influenced the specificity in predicting HIVDR.

**Table 3:** Optimizing the threshold of hair 3TC concentration and comparing it with self-reported adherence among HIV patients with viral load≥1000copies/ml and HIVDR (N = 79).

	With HIVDR	Without HIVDR	Sensitivity	Specificity	PPV	NPV	Youden Index
Hair 3TC≥100	29	14					
<100	11	25	72.5%	64.1%	67.4%	69.4%	36.6%
Hair 3TC≥180	28	10					
<180	12	29	70.0%	74.4%	73.7%	70.7%	44.4%
Hair 3TC≥300	27	9					
<300	13	30	67.5%	76.9%	75.0%	69.8%	44.4%
Hair 3TC≥400	24	9					
<400	16	30	60.0%	76.9%	72.7%	65.2%	36.9%
Missed dose in past month							
No	29	24					
Yes	11	15	72.5%	38.5%	54.7%	57.7%	11.0%
Ratio (90%) of on-time drug intake in the past month							
No	34	26					
Yes	6	13	85.0%	33.3%	56.7%	68.4%	18.3%

## Discussion

In our study, we first demonstrated that 3TC concentrations in hair are significantly different among groups of virologic suppression, virologic failure without HIVDR, and virologic failure with HIVDR. Patients who had a suppressed viral load had the highest hair concentration, illustrating their favorable adherence. Intermediate concentrations in virologic failure patients were associated with the emergence of drug resistance mutations. This may be because of irregular missed doses or non-adherence [16, 32].

In addition, we also found that hair 3TC concentrations have a strong association with virologic outcomes and are able to give a comparatively accurate prediction of virologic outcomes. According to a previous report conducted by Gandhi [11], the odds ratio for virologic suppression was 39.8 for those with hair LPV/r levels in the top tertile compared to the lowest tertile, and for patients with ATV, the adjusted OR was 7.7. Another study of hair NVP concentrations reported that the odds ratio for viral suppression in the highest concentration tertile was 9.17 compared to the lowest tertile [18]. Other studies also reported the sensitivity and specificity of predicting virologic outcome with drug concentrations in hair. A study of the IDV concentration in hair reported that the sensitivity and specificity of predicting virologic outcomes were 75% and 78%, respectively [16]. Another study concluded that the LPV/r concentration in hair had a PPV of 79% and a NPV of 89% [17]. Our study found that the hair 3TC concentration is reliable in assessing patients’ adherence. However, there were two reasons that may limit the sensitivity and specificity. First, patients with HIVDR had a large amount of false-negatives (patients who had high hair 3TC concentrations while experiencing virologic failure at the same time) which reduced the sensitivity. Second, HAART is always a combined therapy and the detection of one drug may reduce the specificity.

According to our previous studies, self-reported adherence variables of the questionnaire were significantly associated with HIV virologic failure and/or HIVDR, including missed doses and the ratio of on-time drug intake in the past month [33, 34]. The self-report adherence variables were suggested by the WHO HIVDR surveillance protocol that was particularly designed for resource-limited settings [35]. What is more, the self-report questionnaires are quite simple and less costly compared with other methods. However, the sensitivity of self-reports was still low and proved that self-report questionnaires would overestimate the adherence. The results may be related to social desirability and recall bias [36]. Our study found that the hair 3TC concentration was a stronger indicator than the self-reported adherence questionnaires in predicting virologic outcomes and HIVDR. Also, hair collection is easy and noninvasive and the storage is less limiting [37]. Other studies showed that plasma 3TC concentrations tend to largely vary among patients and there is no consensus of whether the plasma concentration can predict treatment response [22, 24]. Based on our study, hair 3TC concentrations could be a better measurement of the adherence monitoring index.

The limitations of our study were: first, our sample recruitment from only three provinces could not guarantee a representative sample of all HIV patients in China. The size was not large enough and did not include all kinds of patients in China, which may have lowered the accuracy of the threshold value. Second, most of the participants had been treated for a long-time which could lead to more patients with HIVDR, while hair 3TC concentrations may only reflect the ART treatment adherence in the past month. The relationship between hair 3TC concentrations and HIVDR might be underestimated. Third, data collection did not include any pharmacokinetic related indicators, which may influence the association between hair 3TC concentrations and viral load. We performed this study in a cross-section manner and a longitudinal assessment is required and will be done in the future in order to show causality.

However, these limitations should not affect our conclusion that hair 3TC concentrations can strongly predict virologic outcomes in HIV patients and are a better indicator than self-reported adherence questionnaires in predicting virologic outcomes and HIVDR. Testing lamivudine levels in hair is a simple and safe method for evaluating the ART adherence over a period of months in resource-limited settings. China should establish a centralized unit of drug resistance surveillance to monitor ARV adherence through testing hair concentration of drugs in HIV patients.

## Supporting Information

S1 Table The impact factors of virologic failure. (DOCX)

S2 Table Stratified analysis to explore factors influencing the association between viral loads and hair 3TC concentrations. (DOCX)

S3 Table Stratified analysis of factors which may influence the association between the hair 3TC concentration and the emergence of drug resistance. (DOCX)

## References

[1] Fletcher CV. Pharmacologic considerations for therapeutic success with antiretroviral agents. *Ann Pharmacother* 1999,33:989–995. 10492504 10.1345/aph.19075

[2] Friedland GH, Williams A. Attaining higher goals in HIV treatment: the central importance of adherence. *AIDS* 1999,13 Suppl 1:S61–72. 10546786

[3] Marcellin F, Spire B, Carrieri MP, Roux P. Assessing adherence to antiretroviral therapy in randomized HIV clinical trials: a review of currently used methods. *Expert Rev Anti Infect Ther* 2013,11:239–250. 10.1586/eri.13.8 23458765

[4] Gandhi M, Greenblatt RM. Hair it is: the long and short of monitoring antiretroviral treatment. *Ann Intern Med* 2002,137:696–697. 12379072 10.7326/0003-4819-137-8-200210150-00016

[5] Amico KR, Fisher WA, Cornman DH, Shuper PA, Redding CG, Konkle-Parker DJ, et al Visual analog scale of ART adherence: association with 3-day self-report and adherence barriers. *J Acquir Immune Defic Syndr* 2006,42:455–459. 16810111 10.1097/01.qai.0000225020.73760.c2

[6] Arnsten JH, Demas PA, Farzadegan H, Grant RW, Gourevitch MN, Chang CJ, et al Antiretroviral therapy adherence and viral suppression in HIV-infected drug users: comparison of self-report and electronic monitoring. *Clin Infect Dis* 2001,33:1417–1423. 11550118 10.1086/323201 PMC2692641

[7] Liu H, Golin CE, Miller LG, Hays RD, Beck CK, Sanandaji S, et al A comparison study of multiple measures of adherence to HIV protease inhibitors. *Ann Intern Med* 2001,134:968–977. 11352698 10.7326/0003-4819-134-10-200105150-00011

[8] Berg KM, Arnsten JH. Practical and conceptual challenges in measuring antiretroviral adherence. *J Acquir Immune Defic Syndr* 2006,43 Suppl 1:S79–87. 17133207 10.1097/01.qai.0000248337.97814.66 PMC2866146

[9] Duong M, Piroth L, Peytavin G, Forte F, Kohli E, Grappin M, et al Value of patient self-report and plasma human immunodeficiency virus protease inhibitor level as markers of adherence to antiretroviral therapy: relationship to virologic response. *Clin Infect Dis* 2001,33:386–392. 11438909 10.1086/321876

[10] Kumar AK, Ramachandran G, Kumar P, Kumaraswami V, Swaminathan S. Can urine lamivudine be used to monitor antiretroviral treatment adherence? *MedGenMed* 2006,8:53. 10.1186/1758-2652-8-4-53 PMC1868331 17415333

[11] Gandhi M, Ameli N, Bacchetti P, Gange SJ, Anastos K, Levine A, et al Protease inhibitor levels in hair strongly predict virologic response to treatment. *AIDS* 2009,23:471–478. 10.1097/QAD.0b013e328325a4a9 19165084 PMC2654235

[12] Huang Y, Gandhi M, Greenblatt RM, Gee W, Lin ET, Messenkoff N. Sensitive analysis of anti-HIV drugs, efavirenz, lopinavir and ritonavir, in human hair by liquid chromatography coupled with tandem mass spectrometry. *Rapid Commun Mass Spectrom* 2008,22:3401–3409. 10.1002/rcm.3750 18837069 PMC2669487

[13] Nakahara Y. Hair analysis for abused and therapeutic drugs. *J Chromatogr B Biomed Sci Appl* 1999,733:161–180. 10572981 10.1016/s0378-4347(99)00059-6

[14] Kintz P, Tracqui A, Mangin P. Pharmacological studies on meprobamate incorporation in human beard hair. *Int J Legal Med* 1993,105:283–287. 8471546 10.1007/BF01370386

[15] Graham K, Koren G, Klein J, Schneiderman J, Greenwald M. Determination of gestational cocaine exposure by hair analysis. *JAMA* 1989,262:3328–3330. 2585678

[16] Bernard L, Vuagnat A, Peytavin G, Hallouin MC, Bouhour D, Nguyen TH, et al Relationship between levels of indinavir in hair and virologic response to highly active antiretroviral therapy. *Ann Intern Med* 2002,137:656–659. 12379065 10.7326/0003-4819-137-8-200210150-00009

[17] van Zyl GU, van Mens TE, McIlleron H, Zeier M, Nachega JB, Decloedt E, et al Low lopinavir plasma or hair concentrations explain second-line protease inhibitor failures in a resource-limited setting. *J Acquir Immune Defic Syndr* 2011,56:333–339. 10.1097/QAI.0b013e31820dc0cc 21239995 PMC3073814

[18] Baxi SM, Greenblatt RM, Bacchetti P, Jin C, French AL, Keller MJ, et al Nevirapine Concentration in Hair Samples Is a Strong Predictor of Virologic Suppression in a Prospective Cohort of HIV-Infected Patients. *PLoS One* 2015,10:e0129100 10.1371/journal.pone.0129100 26053176 PMC4460031

[19] Z F. *China Free ART Manual*.1st edn Beijing:. People's Medical Publishing House; 2005.

[20] Zhang F W Y, Wang J et al *China Free ART Manual*. 3nd edn Beijing:. People's Medical Publishing House; 2008.

[21] Xing H, Ruan Y, Hsi JH, Kan W, Liao L, Leng X, et al Reductions in virological failure and drug resistance in Chinese antiretroviral-treated patients due to lamivudine-based regimens, 2003–12. *J Antimicrob Chemother* 2015,70:2097–2103. 10.1093/jac/dkv078 25855758

[22] Hemanth Kumar AK, Ramachandran G, Rajasekaran S, Padmapriyadarsini C, Narendran G, Anitha S, et al Pharmacokinetics of lamivudine & stavudine in generic fixed-dose combinations in HIV-1 infected adults in India. *Indian J Med Res* 2009,130:451–457. 19942751 PMC2853745

[23] Back D, Gatti G, Fletcher C, Garaffo R, Haubrich R, Hoetelmans R, et al Therapeutic drug monitoring in HIV infection: current status and future directions. *AIDS* 2002,16 Suppl 1:S5–37. 12035820 10.1097/00002030-200203001-00002

[24] Minzi O, Mugoyela V, Gustafsson L. Correlation between lamivudine plasma concentrations and patient self-reported adherence to antiretroviral treatment in experienced HIV patients. *Ther Clin Risk Manag* 2011,7:441–446. 10.2147/TCRM.S23625 22162920 PMC3233527

[25] Panhard X, Legrand M, Taburet AM, Diquet B, Goujard C, Mentre F, et al Population pharmacokinetic analysis of lamivudine, stavudine and zidovudine in controlled HIV-infected patients on HAART. *Eur J Clin Pharmacol* 2007,63:1019–1029. 17694300 10.1007/s00228-007-0337-x PMC2703659

[26] Yuen GJ, Lou Y, Bumgarner NF, Bishop JP, Smith GA, Otto VR, et al Equivalent steady-state pharmacokinetics of lamivudine in plasma and lamivudine triphosphate within cells following administration of lamivudine at 300 milligrams once daily and 150 milligrams twice daily. *Antimicrob Agents Chemother* 2004,48:176–182. 14693537 10.1128/AAC.48.1.176-182.2004 PMC310153

[27] Huang Y, Yang Q, Yoon K, Lei Y, Shi R, Gee W, et al Microanalysis of the antiretroviral nevirapine in human hair from HIV-infected patients by liquid chromatography-tandem mass spectrometry. *Anal Bioanal Chem* 2011,401:1923–1933. 10.1007/s00216-011-5278-7 21847531 PMC3477620

[28] Gandhi M, Ameli N, Bacchetti P, Anastos K, Gange SJ, Minkoff H, et al Atazanavir concentration in hair is the strongest predictor of outcomes on antiretroviral therapy. *Clin Infect Dis* 2011,52:1267–1275. 10.1093/cid/cir131 21507924 PMC3079399

[29] Wang X, Yang L, Li H, Zuo L, Liang S, Liu W, et al Factors associated with HIV virologic failure among patients on HAART for one year at three sentinel surveillance sites in China. *Curr HIV Res* 2011,9:103–111. 21361864 10.2174/157016211795569122

[30] Ruan Y, Xing H, Wang X, Tang H, Wang Z, Liu H, et al Virologic outcomes of first-line HAART and associated factors among Chinese patients with HIV in three sentinel antiretroviral treatment sites. *Trop Med Int Health* 2010,15:1357–1363. 10.1111/j.1365-3156.2010.02621.x 20868414

[31] LeBeau MA, Montgomery MA, Brewer JD. The role of variations in growth rate and sample collection on interpreting results of segmental analyses of hair. *Forensic Sci Int* 2011,210:110–116. 10.1016/j.forsciint.2011.02.015 21382678

[32] Condra JH. Resisting resistance: maximizing the durability of antiretroviral therapy. *Ann Intern Med* 1998,128:951–954. 9634437 10.7326/0003-4819-128-11-199806010-00017

[33] Leng X, Liang S, Ma Y, Dong Y, Kan W, Goan D, et al HIV virological failure and drug resistance among injecting drug users receiving first-line ART in China. *BMJ Open* 2014,4:e005886 10.1136/bmjopen-2014-005886 25319999 PMC4202012

[34] Xing H, Ruan Y, Li J, Shang H, Zhong P, Wang X, et al HIV drug resistance and its impact on antiretroviral therapy in Chinese HIV-infected patients. *PLoS One* 2013,8:e54917 10.1371/journal.pone.0054917 23405098 PMC3566114

[35] WHO global strategy for the surveillance and monitoring of HIV drug resistance 2012. Available: http://www.who.int/hiv/pub/drugresistance/drug_resistance_strategy/en/. Accessed 2014 April 30.

[36] Mannheimer S, Friedland G, Matts J, Child C, Chesney M. The consistency of adherence to antiretroviral therapy predicts biologic outcomes for human immunodeficiency virus-infected persons in clinical trials. *Clin Infect Dis* 2002,34:1115–1121. 11915001 10.1086/339074

[37] Gandhi M, Yang Q, Bacchetti P, Huang Y. Short communication: A low-cost method for analyzing nevirapine levels in hair as a marker of adherence in resource-limited settings. *AIDS Res Hum Retroviruses* 2014,30:25–28. 10.1089/AID.2013.0239 24164410 PMC3887402

