# Supplementary material for: Lamivudine Concentration in Hair and Prediction of Virologic Failure and Drug Resistance among HIV Patients Receiving Free ART in China
Source: PLoS One. 2016 Apr 27;11(4):e0154421. doi: 10.1371/journal.pone.0154421 (PMC4847920; doi:10.1371/journal.pone.0154421)
Supplement: S1 Table — (DOCX) [file pone.0154421.s001.docx]

S1 Table . The impact factors of virologic failure

| Characteristic | N | Virologic suppression  Failure N(%) | Crude OR(95%CI) | P-Value | Adjusted OR(95%CI) | P-Value |
| --- | --- | --- | --- | --- | --- | --- |
| Total | 287 | 79(27.5) |  |  |  |  |
| Sex |  |  |  |  |  |  |
| Male | 151 | 40(26.5) |  |  |  |  |
| Female | 136 | 39(28.7) | 1.1(0.7,1.9) | 0.68 |  |  |
| Marital status |  |  |  |  |  |  |
| Married | 218 | 60(27.5) |  |  |  |  |
| Other | 69 | 19(27.5) | 0.8(0.5,1.4) | 0.43 |  |  |
| Education |  |  |  |  |  |  |
| Primary school or less | 149 | 44(29.5) |  |  |  |  |
| Junior high school or more | 138 | 35(25.4) | 1.0(0.5,1.8) | 0.99 |  |  |
| Occupation |  |  |  |  |  |  |
| Farmer | 196 | 56(28.6) |  |  |  |  |
| Others | 91 | 23(25.3) | 0.8(0.5,1.5) | 0.56 |  |  |
| HIV transmission route |  |  |  |  |  |  |
| Former plasma donation | 203 | 56(27.6) |  |  |  |  |
| Other | 84 | 23(27.4) | 0.9(0.6,1.8) | 0.97 |  |  |
| Initial ART regimen |  |  |  |  |  |  |
| Initial regimen with 3TC | 155 | 48(31.0) |  |  |  |  |
| Initial regimen without 3TC | 132 | 31(23.5) | 0.7(0.4,1.2) | 0.16 |  |  |
| Current ART regimen |  |  |  |  |  |  |
| First-line regimen | 151 | 30(19.9) |  |  |  |  |
| Second-line regimen | 136 | 49(36.0) | 2.3(1.3,3.9) | <0.01 |  |  |
| Duration of ART(mean±SD, month) |  |  |  |  |  |  |
| <36 month | 71 | 24(33.8) |  |  |  |  |
| 36-95.9 month | 51 | 11(21.6) | 0.5(0.2,1.2) | 0.14 |  |  |
| ≥96 month | 165 | 44(26.7) | 0.7(0.4,1.3) | 0.27 |  |  |
| Missed doses in the past month |  |  |  |  |  |  |
| No | 228 | 53(23.3) |  |  |  |  |
| Yes | 59 | 26(44.1) | 2.6(1.4,4.7) | <0.01 |  |  |
| Ratio of on-time drug intake in the past month |  |  |  |  |  |  |
| 90% or more | 244 | 60(24.6) |  |  |  |  |
| 89% or less | 43 | 19(44.2) | 2.4(1.2,4.7) | <0.01 |  |  |
| CD4 cell count (cells/mm^3^) at the survey |  |  |  |  |  |  |
| <200 | 44 | 25(56.8) |  |  |  |  |
| 200-349 | 78 | 31(39.7) | 0.5(0.2,1.1) | 0.07 | 0.5(0.2,1.1) | 0.07 |
| ≥350 | 165 | 23(13.9) | 0.1(0.05,0.3) | <0.01 | 0.1(0.05,0.3) | <0.01 |
| Hair 3TC concentration |  |  |  |  |  |  |
| ≥260ng/g | 223 | 36(16.1) |  |  |  |  |
| <260ng/g | 64 | 43(67.2) | 10.6(5.7,20.0) | <0.01 | 11.5(5.7,23.2) | <0.01 |
