# Supplementary material for: Lamivudine Concentration in Hair and Prediction of Virologic Failure and Drug Resistance among HIV Patients Receiving Free ART in China
Source: PLoS One. 2016 Apr 27;11(4):e0154421. doi: 10.1371/journal.pone.0154421 (PMC4847920; doi:10.1371/journal.pone.0154421)
Supplement: S2 Table — (DOCX) [file pone.0154421.s002.docx]

S2 Table . Stratified analysis to explore factors influencing the association between viral loads and hair 3TC concentrations

|  | N | Sensitivity | P | Specificity | P | PPV | P | NPV | P |
| --- | --- | --- | --- | --- | --- | --- | --- | --- | --- |
| Province |  |  |  |  |  |  |  |  |  |
| Henan & Anhui | 181 | 76.7% |  | 91.4% |  | 63.9% |  | 95.2% |  |
| Zhejiang | 66 | 77.8% | 1.00 | 86.0% | 0.25 | 46.7% | 0.25 | 96.1% | 1.00 |
| Age |  |  |  |  |  |  |  |  |  |
| <45 | 126 | 88.2% |  | 93.6% |  | 68.2% |  | 98.1% |  |
| ≥45 | 121 | 68.2% | 0.25 | 85.9% | 0.07 | 51.7% | 0.24 | 92.4% | 0.09 |
| Sex |  |  |  |  |  |  |  |  |  |
| Male | 132 | 81.0% |  | 89.2% |  | 58.6% |  | 96.1% |  |
| Female | 115 | 72.2% | 0.71 | 90.7% | 0.71 | 59.1% | 0.97 | 94.6% | 0.74 |
| Marital status |  |  |  |  |  |  |  |  |  |
| Married | 188 | 73.3% |  | 91.1% |  | 61.1% |  | 94.7% |  |
| Others | 59 | 88.9% | 0.65 | 86.0% | 0.29 | 53.3% | 0.61 | 97.7% | 0.69 |
| Education |  |  |  |  |  |  |  |  |  |
| Junior high school or less | 201 | 75.8% |  | 90.5% |  | 61.0% |  | 95.0% |  |
| High school or more | 46 | 83.3% | 1.00 | 87.5% | 0.56 | 50.0% | 0.72 | 97.2% | 1 |
| Occupation |  |  |  |  |  |  |  |  |  |
| Farmer | 171 | 77.4% |  | 91.4% |  | 66.7% |  | 94.8% |  |
| Others | 76 | 75.0% | 1.00 | 86.8% | 0.30 | 40.0% | 0.72 | 96.7% | 0.08 |
| HIV transmission route |  |  |  |  |  |  |  |  |  |
| Blood Donation | 177 | 76.7% |  | 91.2% |  | 63.9% |  | 95.0% |  |
| Sexual intercourse | 62 | 75.0% |  | 85.2% |  | 42.9% |  | 95.8% |  |
| Others | 8 | 100.0% | 1.00 | 100.0% | 0.31 | 100.0% | 0.21 | 100.0% | 1.00 |
| Treatment duration |  |  |  |  |  |  |  |  |  |
| <36 month | 57 | 80.0% |  | 80.9% |  | 47.1% |  | 95.0% |  |
| 36-96 month | 47 | 100.0% |  | 95.0% |  | 77.8% |  | 100.0% |  |
| ≥96 month | 143 | 68.2% | 0.26 | 91.7% | 0.06 | 60.0% | 0.31 | 94.1% | 0.39 |
| CD4 count during treatment |  |  |  |  |  |  |  |  |  |
| <200 | 28 | 77.8% |  | 89.5% |  | 77.8% |  | 89.5% |  |
| 200-350 | 66 | 73.7% |  | 91.5% |  | 77.8% |  | 89.6% |  |
| ≥350 | 153 | 81.8% | 1.00 | 89.4% | 0.94 | 37.5% | 0.01 | 98.4% | 0.01 |
| Initial ART |  |  |  |  |  |  |  |  |  |
| With 3TC | 131 | 91.7% |  | 88.8% |  | 64.7% |  | 97.9% |  |
| Without 3TC | 116 | 53.3% | 0.02 | 91.1% | 0.58 | 47.1% | 0.23 | 92.9% | 0.17 |
| Current ART |  |  |  |  |  |  |  |  |  |
| First-line ART | 136 | 86.7% |  | 89.3% |  | 50.0% |  | 98.2% |  |
| Second- line ART | 111 | 70.8% | 0.44 | 90.8% | 0.71 | 68.0% | 0.19 | 91.9% | 0.04 |
