# Supplementary material for: Lamivudine Concentration in Hair and Prediction of Virologic Failure and Drug Resistance among HIV Patients Receiving Free ART in China
Source: PLoS One. 2016 Apr 27;11(4):e0154421. doi: 10.1371/journal.pone.0154421 (PMC4847920; doi:10.1371/journal.pone.0154421)
Supplement: S3 Table — (DOCX) [file pone.0154421.s003.docx]

S3 Table . Stratified analysis of factors which may influence the association between the hair 3TC concentration and the emergence of drug resistance

|  | N | Sensitivity | P | Specificity | P | PPV | P | NPV | P |
| --- | --- | --- | --- | --- | --- | --- | --- | --- | --- |
| Province |  |  |  |  |  |  |  |  |  |
| Henan & Anhui | 58 | 71.40% |  | 76.70% |  | 74.10% |  | 74.20% |  |
| Zhejiang | 21 | 66.70% | 1.00 |  | 0.67 | 72.70% | 1.00 | 60.00% | 0.44 |
| Age |  |  |  |  |  |  |  |  |  |
| <45 | 43 | 76.20% |  | 63.60% |  | 66.70% |  | 73.70% |  |
| ≥45 | 36 | 63.20% | 0.37 | 88.20% | 0.14 | 85.70% | 0.27 | 68.20% | 0.7 |
| Sex |  |  |  |  |  |  |  |  |  |
| Male | 40 | 73.70% |  | 81.00% |  | 77.80% |  | 77.30% |  |
| Female | 39 | 66.70% | 0.63 | 66.70% | 0.46 | 70.00% | 0.72 | 63.20% | 0.32 |
| Marital status |  |  |  |  |  |  |  |  |  |
| Married | 60 | 70.00% |  | 73.30% |  | 72.40% |  | 71.00% |  |
| Others | 19 | 70.00% | 1.00 | 77.80% | 1.00 | 77.80% | 1.00 | 70.00% | 1.00 |
| Education |  |  |  |  |  |  |  |  |  |
| Junior high school or less | 63 | 73.30% |  | 72.70% |  | 71.00% |  | 75.00% |  |
| High school or more | 16 | 60.00% | 0.45 | 83.30% | 1.00 | 85.70% | 0.65 | 55.60% | 0.41 |
| Occupation |  |  |  |  |  |  |  |  |  |
| Farmer | 56 | 68.00% |  | 77.40% |  | 70.80% |  | 75.00% |  |
| Other | 23 | 73.30% | 1.00 | 62.50% | 0.40 | 78.60% | 0.72 | 55.60% | 0.41 |
| HIV transmission route |  |  |  |  |  |  |  |  |  |
| Blood Donation | 56 | 73.10% |  | 76.70% |  | 73.10% |  | 76.70% |  |
| Sexual intercourse and others | 23 | 64.30% | 0.72 | 66.70% | 0.67 | 75.00% | 1.00 | 54.50% | 0.25 |
| Treatment duration |  |  |  |  |  |  |  |  |  |
| ＜36M | 24 | 64.30% |  | 80.00% |  | 81.80% |  | 61.50% |  |
| 36-96M | 11 | 75.00% |  | 85.70% |  | 75.00% |  | 85.70% |  |
| ≥96M | 34 | 72.70% | 0.88 | 41.70% | 0.69 | 69.60% | 0.86 | 45.50% | 0.53 |
| CD4 count during treatment |  |  |  |  |  |  |  |  |  |
| <200 | 25 | 68.80% |  | 77.80% |  | 84.60% |  | 58.30% |  |
| 200-350 | 31 | 66.70% |  | 73.70% |  | 61.50% |  | 77.80% |  |
| ≥350 | 23 | 75.00% | 1.00 | 72.70% | 1.00 | 75.00% | 0.43 | 72.70% | 0.57 |
| Initial ART |  |  |  |  |  |  |  |  |  |
| With 3TC | 48 | 66.70% |  | 87.50% |  | 84.20% |  | 72.40% |  |
| Without 3TC | 31 | 75.00% | 0.73 | 53.30% | 0.03 | 63.20% | 0.14 | 66.70% | 0.72 |
| Current ART |  |  |  |  |  |  |  |  |  |
| First-line ART | 30 | 66.70% |  | 80.00% |  | 76.90% |  | 70.60% |  |
| Second- or third-line | 49 | 72.00% | 0.74 | 70.80% | 0.71 | 72.00% | 1.00 | 70.80% | 1.00 |
